# N-6-Adenine-Specific DNA Methyltransferase 1 (*N6AMT1*) Polymorphisms and Arsenic Methylation in Andean Women

**DOI:** 10.1289/ehp.1206003

**Published:** 2013-05-10

**Authors:** Florencia Harari, Karin Engström, Gabriela Concha, Graciela Colque, Marie Vahter, Karin Broberg

**Affiliations:** 1 Institute of Environmental Medicine, Unit of Metals and Health, Karolinska Institutet, Stockholm, Sweden; 2 Department of Laboratory Medicine, Section of Occupational and Environmental Medicine, Lund University, Lund, Sweden; 3 Swedish National Food Agency, Department of Risk-Benefit Assessment, Uppsala, Sweden; 4 Hospital Dr. Nicolás Cayetano Pagano, San Antonio de los Cobres, Argentina

**Keywords:** DMA, gene × environment interactions, inorganic arsenic, methylation, MMA, polymorphisms

## Abstract

Background: In humans, inorganic arsenic is metabolized to methylated metabolites mainly by arsenic (+3 oxidation state) methyltransferase (AS3MT). *AS3MT* polymorphisms are associated with arsenic metabolism efficiency. Recently, a putative N-6-adenine-specific DNA methyltransferase 1 (N6AMT1) was found to methylate arsenic *in vitro*.

Objective: We evaluated the role of *N6AMT1* polymorphisms in arsenic methylation efficiency in humans.

Methods: We assessed arsenic methylation efficiency in 188 women exposed to arsenic via drinking water (~ 200 µg/L) in the Argentinean Andes by measuring the relative concentrations of arsenic metabolites in urine [inorganic arsenic, methylarsonic acid (MMA), and dimethylarsinic acid] by high-performance liquid chromatography coupled with hydride generation and inductively coupled plasma mass spectrometry. We performed genotyping for *N6AMT1* and *AS3MT* polymorphisms by Taqman assays, and gene expression (in blood; *n* = 63) with Illumina HumanHT-12 v4.0.

Results: Five *N6AMT1* single nucleotide polymorphisms (SNPs; rs1997605, rs2205449, rs2705671, rs16983411, and rs1048546) and two *N6AMT1* haplotypes were significantly associated with the percentage of MMA (%MMA) in urine, even after adjusting for *AS3MT* haplotype. %MMA increased monotonically according to the number of alleles for each SNP (e.g., for rs1048546, mean %MMA was 7.5% for GG, 8.8% for GT, and 9.7% for TT carriers). Three SNPs were in linkage disequilibrium (*R*^2^ > 0.8). Estimated associations for joint effects of *N6AMT1* (haplotype 1) and *AS3MT* (haplotype 2) were generally consistent with expectations for additive effects of each haplotype on %MMA. Carriers of *N6AMT1* genotypes associated with lower %MMA showed the lowest *N6AMT1* expression, but associations were monotonic according to copy number for only one genotype and one haplotype.

Conclusions: *N6AMT1* polymorphisms were associated with arsenic methylation in Andean women, independent of *AS3MT*. *N6AMT1* polymorphisms may be susceptibility markers for arsenic-related toxic effects.

Inorganic arsenic (iAs) is a toxic and carcinogenic metalloid found at high concentrations in drinking water in many regions around the world [International Agency for Research on Cancer (IARC) 2004]. As a consequence, millions of persons are continuously exposed to arsenic. Individuals who are exposed to iAs in water or food metabolize arsenic by a series of methylation reactions, converting iAs to mono- (MMA) and dimethylated (DMA) metabolites, which are excreted in urine (Marapakala et al. 2012; Vahter 2002). There are major differences in the efficiency of arsenic methylation between individuals and population groups, and less-efficient arsenic metabolism is associated with the increased incidence of arsenic-related health effects such as skin lesions, cardiovascular disease, diabetes, and cancer (Chung et al. 2002; IARC 2004; Leonardi et al. 2012; Lindberg et al. 2008b; Mazumder et al. 2005; Meliker et al. 2007; Navas-Acien et al. 2005). Thus, it is essential to elucidate the factors influencing arsenic metabolism and susceptibility.

Evaluation of the efficiency of arsenic methylation is mainly based on the relative amounts of the different metabolites in urine. Typically, efficient metabolizers of arsenic have > 80% of the total arsenic in urine in the form of DMA and have low percentages of iAs and MMA (Gardner et al. 2011; Lindberg et al. 2008a; Vahter 2002). Poor metabolizers usually have < 60% of the total arsenic in urine as DMA, 20–30% as MMA, and 10–30% as iAs (Vahter 2002).

*S*-Adenosylmethionine is the main methyl donor for arsenic (Marafante and Vahter 1984). The methyl transfer is accomplished by methyltransferases, in humans mainly the arsenic (+3 oxidation state) methyltransferase (AS3MT) (Engström et al. 2011). Polymorphisms in *AS3MT* contribute substantially to the variability in the pattern of excreted arsenic metabolites in different populations (Engström et al. 2011; Meza et al. 2005). However, *As3mt* knockout mice are still capable of methylating arsenic, albeit to a lesser extent, suggesting the existence of alternative methylation pathways (Chen et al. 2011; Drobna et al. 2009). There is also evidence that other methyltransferases, such as DNA-methyltransferases (DNMT1a, DNMT3b), are capable of arsenic methylation (Engström et al. 2011).

In a recent study, Jo et al. (2009) screened yeast deletion mutants to identify genes required for the growth of yeast in the presence of MMAIII and iAsIII and found that an *S*-adenosylmethionine–dependent methyltransferase, corresponding to the putative N-6-adenine-specific DNA methyltransferase 1 (*N6AMT1*) in humans, conferred resistance to these arsenic species. In addition, Ren et al. (2011) showed that N6AMT1 could metabolize arsenic in human urothelial cells *in vitro* and that N6AMT1 selectively metabolized MMA to DMA. The objective of the present study was to assess associations between polymorphisms in *N6AMT1* and the efficiency of human arsenic methylation.

## Materials and Methods

*Study area and population*. Participants were all women (*n* = 188) living on the Andean plateau (~ 3,800 m above sea level) in northern Argentina and exposed to varying levels of arsenic from their drinking water (Concha et al. 2010). The study area has minimal industrial or traffic pollution. Women were recruited in 2008 and 2011 from the village of San Antonio de los Cobres [water arsenic ~ 200 µg/L (Concha et al. 2010)], with about 6,000 inhabitants, and from small surrounding villages with lower arsenic exposure [see Supplemental Material, Table S1 (http://dx.doi.org/10.1289/ehp.1206003)], with water arsenic levels between 3.5 and 70 µg/L. In 2008 we recruited 154 women (137 from San Antonio de los Cobres and 17 from the surrounding villages), and in 2011 we recruited 34 women (17 from San Antonio de los Cobres and 17 from the surrounding villages). The women recruited in San Antonio de los Cobres in 2008 were significantly older than the women from 2011 (mean, 38 vs. 31 years of age). Apart from that, there were no significant differences in exposure or main characteristics between the groups (see Supplemental Material, Table S2). We recruited the study participants with the assistance of medical personnel from the main hospital. Most of the women were accompanying their relatives to the hospital or health care centers. In the small village of Tolar Grande, however, we went from house to house, explained the project, and invited adult women to participate. We included only nonpregnant women because pregnancy has been shown to influence arsenic methylation (Gardner et al. 2012). Concerning selection of the participants, besides pregnancy, we asked if women lived permanently in the villages where the study was performed. Women who participated in the 2008 study were not eligible for recruitment in 2011. In addition, women who were first-degree relatives of other participants in the study, recruited in 2008 or 2011, were excluded. No other inclusion/exclusion criteria were used. Men are often away from home at work for most of the day and drink water from different sources; thus, it is difficult to evaluate their actual exposure. In further contrast with the men, women in this society rarely drink alcohol or smoke (Engström et al. 2011): Only 7 (3.7%) of the study participants smoked cigarettes and none reported drinking alcohol. Therefore, to decrease the influence of factors such as smoking and alcohol and to accurately estimate arsenic exposure, men were not included in this study. Almost half of the women (53%) reported that they currently chewed coca leaves.

This study group was chosen because they have a large range in arsenic exposure and they are efficient arsenic metabolizers compared with other populations (Engström et al. 2011; Vahter 2002).

The women were interviewed about their personal characteristics, including age, parity, ancestry, drinking water sources, current dietary habits, ongoing and previous diseases, and time of residence in the study area. The same questionnaire was used in 2008 and 2011. Also, weight and height were measured, and body mass index (BMI; weight in kilograms divided by squared height in meters) was calculated.

We collected blood and urine samples from each individual in the same way in 2008 and 2011. Venous blood samples were collected in K_2_EDTA tubes (Vacuette®; Greiner Bio-One GmbH, Greiner, Germany) for DNA extraction and PAX tubes (PreAnalytiX GmbH, Hombrechtikon, Switzerland) for gene expression analyses. Spot urine samples were collected in disposable urine collection cups and immediately transferred to 20-mL polyethylene bottles. We collected all the biological samples at the hospital or the local health clinics during the daytime; the project logistics did not allow for fasting before sampling. After a maximum of 24 hr at room temperature after sampling, blood and urine samples were frozen and kept at –20°C until they were transported with cooling blocks to Sweden for analyses. Analyses took place within 2 months of collection.

Both oral and written informed consents were provided by all the study participants. The study was approved by the Ministry of Health in Salta, Argentina, and the Regional Ethical Committee at Karolinska Institutet.

*Arsenic exposure and metabolism*. We assessed exposure to iAs based on the sum of the concentrations of the inorganic arsenic metabolites (iAs + MMA + DMA) in urine (U-As), and we assessed the efficiency of arsenic metabolism based on the relative proportions (percentage of the sum of urinary arsenic metabolites) of iAs metabolites (iAsIII, iAsV), MMA, and DMA in urine (Vahter 2002). Arsenic metabolites in urine (i.e., iAs, MMA, and DMA) were determined using high-performance liquid chromatography (HPLC) (Agilent 1100 series system; Agilent Technologies, Waldbronn, Germany) coupled with hydride generation (HG) and inductively coupled plasma mass spectrometry (ICP-MS: Agilent 7500ce; Agilent Technologies, Tokyo, Japan) (Concha et al. 2010). The HG system was used to introduce only the metabolites of iAs to the ICP-MS. After shaking the urine samples, approximately 0.5 mL of each urine sample was filtered on a 0.20-μm syringe filter and transferred to the HPLC-HG-ICPMS system. For quality control, we analyzed the reference material CRM No.18 (certified DMA concentration of 36 ± 9 µg/L; National Institute for Environmental Studies, Ibaraki, Japan) along with the collected samples. We obtained 43.9 ± 4.7 µg/L (mean ± SD; *n* = 21), which agreed with previously reported results (Li et al. 2008). In order to compensate for variation in urine dilution, we adjusted the measured concentrations of arsenic in urine to the mean specific gravity of urine (1.020 g/mL), determined by a digital refractometer (EUROMEX RD 712 clinical refractometer; EUROMEX, Arnhem, the Netherlands).

*Genotyping and gene expression analysis.* DNA was isolated from peripheral blood with the Qiagen DNA Blood Mini kit (QIAGEN, Hilden, Germany). We genotyped five *N6AMT1* single nucleotide polymorphisms (SNPs in 5´–3´ order: rs1997605, rs2205449, rs2705671, rs16983411, and rs1048546) by Taqman® SNP genotyping assays on a fast real-time PCR System (7900HT; Applied Biosystems, Carlsbad, CA, USA) according to the manufacturer’s instructions. Blanks and controls for each genotype were included in each run, and genotyping was repeated on 5% of the samples.

Because none of the *N6AMT1* SNPs have previously been analyzed in relation to functional impact on gene expression or protein activity, we selected SNPs that tag genetic variation within the gene due to strong linkage disequilibrium with other SNPs in the same region (tagSNPs) (Table 1). TagSNPs for *N6AMT1* were selected using Haploview (version 4.1; Barrett et al. 2005) on the HapMap (http://hapmap.ncbi.nlm.nih.gov) CEU population (Utah residents with ancestry from northern and western Europe). We inferred haplotypes from *N6AMT1* rs1997605, rs2205449, rs2705671, and rs1048546 by PHASE software (Stephens and Donnelly 2003). We did not include rs16983411 in the final haplotype analysis because the frequency was very low (minor allele frequency of 1%).

**Table 1:** Allele frequencies of SNPs in putative N-6 adenine-specific DNA methyl­transferase 1 (*N6AMT1*).

SNP^*b*^	SNP type^*b*^	Base^*c*^	Position^*b*^	*n*	Andean study population	HapMap allele frequencies^*a*^ (%)		
					Allele frequency (%)^*d*^	European	Asian	African
rs1997605	Intron 1	A/G	30257418	188	53/47	85/15	50/50	65/35
rs2205449	Intron 4	A/T	30252088	188	38/63	59/41	25/75	18/82
rs2705671	Intron 4	T/G	30251349	184	58/42	86/14	69/31	94/6
rs16983411	Intron 5	A/G	30250247	185	99/1	84/16	87/13	82/18
rs1048546	3´UTR	G/T	30244877	187	53/47	69/31	37/63	59/41
^***a***^Allele frequencies for Europeans, Asians, and Africans from HapMap CEU (CEPH, Utah residents with ancestry from northern and western Europe), JPT (Japanese in Tokyo, Japan), and YRI (Yoruba in Ibadan, Nigeria), respectively (Thorisson etal. 2005). ^***b***^From the NCBI dbSNP Single Nucleotide Polymorphisms database (http://www.ncbi.nlm.nih.gov/SNP), genome build 37.3. ^***c***^Alleles associated with lower %MMA are denoted first. ^***d***^Chi-square values from the test of Hardy Weinberg equilibrium were 2.73 for rs1997605, 0.41 for rs2205449, 3.21 for rs2705671, <0.001 for rs16983411, and 2.11 for rs1048546.								

Genotyping for *AS3MT* SNPs associated with arsenic metabolism was performed as described previously (Engström et al. 2011). Briefly, eight SNPs were genotyped using Sequenom™ (Sequenom, San Diego, CA, USA) technology. We inferred haplotypes from the *AS3MT* SNPs by PHASE software. In this population we previously found that the major *AS3MT* haplotype (referred to here as haplotype 2; haplotype frequency = 70%) was GCCATCAC [5´–3´ order of *AS3MT* SNPs: rs7085104, rs3740400, rs3740393, rs3740390, rs11191439, rs11191453, rs10748835, and rs1046778 (Engström et al. 2011)]. This haplotype was associated with low %MMA and high %DMA, consistent with more efficient arsenic metabolism (Engström et al. 2011). In the 34 women recruited in 2011, we genotyped *AS3MT* rs3740400, rs3740393, rs11191439, and rs1046778 with Taqman® SNP genotyping assays according to the manufacturer’s instructions, and inferred haplotype 2 based on the four SNPs only, as we have shown that we obtain very similar inferred *AS3MT* haplotypes with fewer SNPs compared with a larger number of SNPs (Schlebusch et al. 2013).

To examine gene expression we extracted RNA with the PAXgene Blood RNA kit (PreAnalytiX) and stored the samples at –80°C. We evaluated RNA concentration and purity using a Nanodrop spectrophotometer (NanoDrop Technologies, Wilmington, DE, USA) and RNA integrity with a Bioanalyzer 2100 (Agilent Technologies, Santa Clara, CA, USA), and the results confirmed high-quality RNA (RNA integrity number > 7.5, where 1 is the worst and 10 the best). For the gene expression analysis, we selected participants who had the best quality and highest quantity of RNA for array analyses. These individuals, with a wide range of urinary arsenic concentrations (10–1,251 µg/L), were classified into low/high U-As groups based on median values, and then frequency-matched for age, weight, and BMI so that there were no major differences between the two groups (*p* > 0.05 for age, weight/BMI), resulting in a total of 63 individuals for the gene expression analyses. For the whole genome gene expression analysis, we used DirectHyb HumanHT-12, version 4.0 (Illumina, San Diego, CA, USA) according to the manufacturer’s instructions, and the analysis was performed at the Swegene Center for Integrative Biology at Lund University. Background signals were filtered by BioArray Software Environment (BASE) (Vallon-Christersson et al. 2009) and results are presented in relative fluorescence units. There were two *N6AMT1* transcripts in the HumanHT-12, version 4.0 array: ILMN_2315569, corresponding to mRNA NM_182749.2, which encodes the longer isoform [National Center of Biotechnology Information (NCBI) Nucleotide database (http://www.ncbi.nlm.nih.gov/nuccore)], and ILMN_1754988, corresponding to NM_013240.3, which lacks an alternate in-frame exon, resulting in a shorter protein.

*Statistical analysis*. We analyzed deviations from Hardy-Weinberg equilibrium using chi-square analysis, and estimated linkage disequilibrium using Haploview (Barrett 2009).

We estimated associations between genotypes or haplotypes (independent variables) and proportions of individual metabolites (%iAs metabolites, %MMA, or %DMA as dependent variables) in multivariable-adjusted regression models. All models were adjusted for natural log–transformed total urinary arsenic concentration (lnU-As) because the level of arsenic exposure in itself influences metabolism (Vahter 2002). Genotypes and haplotypes were modeled as categorical variables (for genotypes: zero, one, or two alleles; and for haplotypes: zero, one, or two copies), with the genotype or haplotype associated with low MMA fractions as the reference group for each variant in all models. When the frequency of a homozygote genotype included < 3% of individuals, this group was pooled with the heterozygotes. In the multivariable-adjusted analyses, we considered the *AS3MT* haplotype to be a potential confounder. We first modeled associations between each SNP genotype or haplotype and each outcome (%iAs, %DMA, or %MMA) in separate models adjusted for lnU-As (model 1), then fit models that also were adjusted for *AS3MT* haplotype (model 2). We also modeled associations between *AS3MT* haplotype 2 and arsenic metabolites adjusted for each *N6AMT1* variant in individual models. In addition, we modeled the joint effects of the most common *N6AMT1* haplotype and *AS3MT* haplotype 2 by estimating associations for each possible combination of genotypes based on the two haplotypes (i.e., with two copies of each haplotype used as the reference category, two copies of the *N6AMT1* haplotype and one copy of the *AS3MT* haplotype, etc., for a total of eight possible combinations), with adjustment for lnU-As.

We analyzed correlations between total arsenic or fraction of MMA in urine and gene expression data using the Spearman correlation coefficient (*r*_S_). Gene expression data were normally distributed and, therefore, relations between *N6AMT1* SNPs/haplotypes and gene expression for *N6AMT1* transcripts were analyzed by analysis of variance (ANOVA).

All calculations were made using IBM SPSS® Statistics, version 20.0 (IBM, Chicago, IL, USA). Statistical significance was determined as *p* < 0.05 (two-tailed).

## Results

*General characteristics*. The characteristics of the study population are presented in Table 2. The studied women (*n* = 188) were on average 34 years of age with a median U-As concentration of 210 µg/L (median urinary fraction of iAs, 12%; MMA, 8.1%; and DMA, 80%).

**Table 2:** Characteristics of the study population.

Characteristic	Median, %, or *n*	5th–95th percentiles
Age [years (median)]	34	19–64
Time of residency [years (median)]	25	3.0–53
Town of residency [SAC/other village (*n*)]^*a*^	154/34
BMI [kg/m^2^ (median)]	25	19–35
Coca users (%)	53
Tobacco users (%)	3.7
Alcohol users (%)	0.0
Total U-As [µg/L (median)]^*b*^	210	33–502
iAs (%)	12	4.8–23
MMA (%)	8.1	4.2–15
DMA (%)	80	65–89
*N6AMT1* haplo­types (*n*)
Haplo­type 1: AATG (2/1/0 copies)^*c*^	30/81/77
Haplo­type 3: ATTG (2/1/0 copies)^*c*^	5/46/137
Haplo­type 9: GTGT (0/1/2 copies)^*c*^	72/73/43
*AS3MT* haplo­type (*n*)
Haplo­type 2: GCCATCAC (2/1/0 copies)^*d*^	90/75/21
^***a***^SAC: San Antonio de los Cobres; other villages: Santa Rosa de los Pastos Grandes, Pocitos, Olacapato, Cobres, Rosario de Lerma. ^***b***^Total U-As was adjusted to the mean specific gravity of 1.020 g/mL. ^***c***^Number of copies associated with lower %MMA are denoted first. ^***d***^Number of copies that have previously been shown to have an association with lower %MMA are denoted first (Engström etal. 2011).		

The *N6AMT1* SNPs were situated within 12,451 base pairs (distance between SNP rs1997605 in intron 1 and rs1048546 in 3´ UTR). Genotypes of all SNPs were in Hardy Weinberg equilibrium (Table 1). The allelic frequencies for the *N6AMT1* SNPs varied between the Andean population and other reference populations from the Hapmap study (Table 1). Rs1048546 was in linkage disequilibrium (LD) with rs2705671 (*R*^2^ = 0.80) and rs1997605 (*R*^2^ = 0.94), and the LD between rs2705671 and rs1997605 was *R*^2^ = 0.85 [see Supplemental Material, Figure S1 (http://dx.doi.org/10.1289/ehp.1206003)]. Rs2205449 was in weaker LD with these three SNPs (*R*^2^ between 0.42 and 0.51). Rs16983411 showed a very low minor allele frequency (1%) in Andeans, and it was not in LD with any of the other SNPs.

*Arsenic metabolite patterns in relation to genotype/haplotype*. At least one genotype of each *N6AMT1* SNP was significantly associated with %MMA in urine based on the multivariable linear regression analysis (Table 3). With the exception of rs16983411, which was modeled as a dichotomous genotype because of small numbers of variant alleles, associations with %MMA increased monotonically according to the number of alleles for all SNPs [see Supplemental Material, Figure S2A–D (http://dx.doi.org/10.1289/ehp.1206003)]. For example, for rs1048546, the mean %MMA was 7.5 [95% confidence interval (CI): 6.7, 8.4] for GG, 8.8 (95% CI: 7.9, 9.4) for GT, and 9.7 (95% CI: 8.9, 10.7) for TT carriers. After adjusting for multiple comparisons (Bonferroni-corrected significance level of 0.01), associations of rs2205449, rs2705671, and rs1048546 with %MMA remained statistically significant. The estimated differences in mean %MMA decreased somewhat after adjusting for *AS3MT* haplotype (model 2, Table 3).

**Table 3:** Multivariable regression analyses^*a*^ of the influence of *N6AMT1* genotypes on fractions of arsenic metabolites.

Metabolite/SNP	Geno­type^*b*^	*n*	Mean (95%CI)^*c*^	Model 1		Model 2	
				β (95%CI)	*p*-Value	β (95%CI)	*p*-Value
iAs
rs1997605	AA	63	13.1 (11.6, 14.6)
	AG	75	12.5 (11.1, 13.9)	–0.60 (–2.7, 1.5)	0.57	–0.69 (–2.7, 1.3)	0.50
	GG	50	12.6 (10.9, 14.3)	–0.50 (–2.8, 1.7)	0.66	–1.1 (–3.3, 1.1)	0.33
rs2205449	AA	30	13.8 (11.7, 16.0)
	TA	81	12.5 (11.2, 13.8)	–1.3 (–3.9, 1.2)	0.30	–1.2 (–3.7, 1.3)	0.34
	TT	77	12.5 (11.1, 13.9)	–1.3 (–3.9, 1.2)	0.30	–1.7 (–4.2, 0.79)	0.18
rs2705671	TT	71	12.8 (11.4, 14.2)
	GT	70	13.1 (11.6, 14.5)	0.27 (–1.8, 2.3)	0.80	–0.047 (–2.0, 1.9)	0.96
	GG	43	12.0 (10.2, 13.9)	–0.76 (–3.1, 1.6)	0.52	–1.3 (–3.5, 1.0)	0.28
rs16983411	AA	181	12.8 (11.9, 13.7)
	AG	4	10.1 (4.1, 16.0)	–2.7 (–8.7, 3.3)	0.38	–3.5 (–9.4, 2.4)	0.24
rs1048546	GG	60	13.2 (11.7, 14.8)
	GT	77	12.5 (11.0, 13.8)	–0.80 (–2.9, 1.3)	0.45	–0.91 (–2.9, 1.1)	0.38
	TT	50	12.6 (10.9, 14.3)	–0.63 (–2.9, 1.6)	0.59	–1.4 (–3.6, 0.88)	0.23
MMA
rs1997605	AA	63	7.7 (7.0, 8.6)
	AG	75	8.6 (7.8, 9.2)	0.67 (–0.44, 1.8)	0.23	0.53 (–0.51, 1.6)	0.32
	GG	50	9.6 (8.9, 10.7)	1.9 (0.74, 3.1)	0.0017	1.5 (0.35, 2.6)	0.011
rs2205449	AA	30	7.2 (6.0, 8.3)
	TA	81	8.6 (7.8, 9.2)	1.3 (–0.070, 2.7)	0.063	1.3 (0.058, 2.6)	0.041
	TT	77	9.2 (8.6, 10.0)	2.1 (0.75, 3.5)	0.0026	1.8 (0.55, 3.1)	0.0054
rs2705671	TT	71	7.7 (7.0, 8.5)
	GT	70	8.7 (7.9, 9.4)	0.84 (–0.24, 1.9)	0.12	0.65 (–0.38, 1.7)	0.21
	GG	43	9.6 (8.8, 10.7)	2.0 (0.73, 3.2)	0.0020	1.6 (0.41, 2.8)	0.0087
rs16983411	AA	181	8.5 (8.0, 9.0)
	AG	4	11.6 (8.6, 15.0)	3.3 (0.063, 6.6)	0.046	2.7 (–0.41, 5.7)	0.089
rs1048546	GG	60	7.5 (6.7, 8.4)
	GT	77	8.8 (7.9, 9.4)	1.1 (0.011, 2.2)	0.048	0.97 (–0.72, 2.0)	0.068
	TT	50	9.7 (8.9, 10.7)	2.3 (1.1, 3.5)	0.00024	1.8 (0.61, 2.9)	0.0031
DMA
rs1997605	AA	63	79.2 (77.3, 80.9)
	AG	75	78.9 (77.4, 80.7)	–0.068 (–2.6, 2.4)	0.96	0.16 (–2.2, 2.5)	0.89
	GG	50	77.8 (75.6, 79.7)	–1.5 (–4.2, 1.3)	0.29	–0.41 (–3.0, 2.2)	0.75
rs2205449	AA	30	79.0 (76.4, 81.6)
	TA	81	79.0 (77.5, 80.7)	0.032 (–3.0, 3.1)	0.98	–0.14 (–3.0, 2.8)	0.92
	TT	77	78.3 (76.6, 79.8)	–0.81 (–3.9, 2.3)	0.60	–0.16 (–3.1, 2.8)	0.92
rs2705671	TT	71	79.5 (77.7, 81.2)
	GT	70	78.4 (76.6, 80.1)	–1.1 (–3.6, 1.4)	0.38	–0.60 (–2.9, 1.7)	0.61
	GG	43	78.2 (76.0, 80.5)	–1.2 (–4.0, 1.6)	0.40	–0.33 (–3.0, 2.3)	0.80
rs16983411	AA	181	78.8 (77.7, 79.8)
	AG	4	78.3 (70.9, 85.3)	–0.64 (–7.9, 6.7)	0.86	0.82 (–6.0, 7.7)	0.81
rs1048546	GG	60	79.4 (77.4, 81.1)
	GT	77	78.8 (77.3, 80.6)	–0.31 (–2.8, 2.2)	0.81	–0.058 (–2.4, 2.3)	0.96
	TT	50	77.8 (75.6, 79.6)	–1.7 (–4.4, 1.1)	0.23	–0.40 (–3.0, 2.2)	0.76
^***a***^Model 1: Arsenic metabolite = α+β×geno­type+γ×U-As (ln transformed). Model 2: Arsenic metabolite =α+β× geno­type+γ×U-As (ln)+λ×*AS3MT* haplo­type. ^***b***^Allele geno­types associated with lower %MMA are denoted first. ^***c***^Mean values and 95% CIs are adjusted values based on model1.							

For each SNP, the direction of associations with %iAs and %DMA were in the opposite direction from associations with %MMA, but were closer to the null and not statistically significant (Table 3). In most cases, the *N6AMT1* genotypes associated with the lowest %MMA were associated with the highest %iAs and %DMA. However, the associations with iAs% and %DMA did not reach statistical significance (Table 3). In general, further adjustment for *AS3MT* increased the estimates for %iAs, which became similar in size to those of %MMA but in the opposite direction, whereas the *AS3MT*-adjustment tended to decrease the estimates for %DMA.

*AS3MT* haplotype 2 was significantly associated with percentages of iAs, MMA, and DMA when adjusted for individual *N6AMT1* SNPs [see Supplemental Material, Table S3 (http://dx.doi.org/10.1289/ehp.1206003)]. Differences in estimated mean %MMA were smaller in association with *N6AMT1* SNPs than in association with *AS3MT* haplotype 2: for example, mean %MMA increased (β = 2.3; 95% CI: 1.1, 3.5) in carriers of rs1048546 TT relative to GG carriers (β = 1.8; 95% CI: 0.61, 2.9 when adjusted for *AS3MT* haplotype) (Table 3), whereas %MMA in women with no copies of *AS3MT* haplotype 2 was increased (β = 3.2; 95% CI: 1.7, 4.6) relative to women with two copies (β = 2.8; 95% CI: 1.3, 4.2 when adjusted for *N6AMT1* rs1048546) (see Supplemental Material, Table S3).

Nine *N6AMT1* haplotypes were inferred from rs1997605, rs2205449, rs2705671, and rs1048546: *a*) AATG, *b*) AAGG, *c*) ATTG, *d*) ATTT, *e*) GAGT, *f*) GTTG, *g*) GTTT, *h*) GTGG, and *i*) GTGG; of which only haplotypes 1, 3, and 9 were common enough (36%, 16%, and 42%, respectively, Table 2) for analyses. %MMA was significantly higher among women with no copies (vs. two copies) of haplotype 1 (β = 2.1; 95% CI: 0.75, 3.5) and among women with two copies (vs. no copies) of haplotype 9 (β = 1.9; 95% CI: 0.72, 3.2) (Table 4). %MMA was also higher among women with no copies (vs. two copies) of haplotype 3, although the association was not statistically significant. The association between %MMA and haplotype 1 remained significant after adjusting for multiple comparisons (Bonferroni-corrected significance level of 0.01). Associations between *N6AMT1* haplotypes and %iAs and %DMA were not statistically significant (Table 4).

**Table 4:** Multivariable regression analyses^*a*^ of the influence of *N6AMT1* haplotypes on fractions of arsenic metabolites.

Metabolite/*N6AMT1* haplo­type (sequence)	*n*	Copy nr^*b*^	Mean (95%CI)^*c*^	Model 1		Model 2	
				β (95%CI)	*p*-Value	β (95%CI)	*p*-Value
iAs
Haplo­type 1 (AATG)	30	2	13.8 (11.7, 16.0)
	81	1	12.5 (11.2, 13.8)	–1.3 (–3.9, 1.2)	0.30	–1.2 (–3.7, 1.3)	0.34
	77	0	12.5 (11.1, 13.9)	–1.3 (–3.9, 1.2)	0.30	–1.7 (–4.2, 0.79)	0.18
Haplo­type 3 (ATTG)	5	2	16.2 (11.0, 21.6)
	46	1	11.7 (10.0, 13.5)	–4.6 (–10, 0.96)	0.10	–4.4 (–9.8, 1.1)	0.18
	137	0	12.9 (11.9, 13.9)	–3.4 (–8.8, 2.0)	0.21	–3.5 (–8.8, 1.8)	0.19
Haplo­type 9 (GTGT)	72	0	12.7 (11.4, 14.2)
	73	1	13.0 (11.6, 14.4)	0.97 (–1.4, 3.3)	0.79	0.020 (–1.9, 2.0)	0.98
	43	2	12.1 (10.3, 13.9)	0.69 (–1.4, 3.3)	0.55	–1.3 (–3.5, 0.98)	0.27
MMA
Haplo­type 1 (AATG)	30	2	7.2 (6.0, 8.3)
	81	1	8.6 (7.8, 9.2)	1.3 (–0.70, 2.7)	0.063	1.3 (0.058, 2.6)	0.041
	77	0	9.2 (8.6, 10.0)	2.1 (0.75, 3.5)	0.003	1.8 (0.55, 3.1)	0.005
Haplo­type 3 (ATTG)	5	2	6.9 (4.3, 10.2)
	46	1	8.4 (7.4, 9.3)	1.1 (–1.9, 4.2)	0.48	1.4 (–1.5, 4.2)	0.35
	137	0	8.7 (8.2, 9.3)	1.5 (–1.5, 4.5)	0.33	1.4 (–1.4, 4.2)	0.32
Haplo­type 9 (GTGT)	72	0	7.8 (7.1, 8.6)
	73	1	8.9 (8.0, 9.5)	0.90 (–0.17, 2.0)	0.098	0.64 (–0.37, 1.6)	0.21
	43	2	9.6 (8.8, 10.7)	1.9 (0.72, 3.2)	0.002	1.5 (0.35, 2.7)	0.011
DMA
Haplo­type 1 (AATG)	30	2	79.0 (76.4, 81.6)
	81	1	79.0 (77.5, 80.7)	0.032 (–3.0, 3.1)	0.98	–0.14 (–3.0, 2.8)	0.92
	77	0	78.3 (76.6, 79.8)	–0.81 (–3.9, 2.3)	0.60	–0.16 (–3.1, 2.8)	0.92
Haplo­type 3 (ATTG)	5	2	77.0 (77.2, 79.6)
	46	1	79.9 (77.8, 82.0)	3.4 (–3.3, 10)	0.32	3.0 (–3.4, 9.3)	0.36
	137	0	78.4 (77.2, 79.6)	1.9 (–4.6, 8.5)	0.57	2.1 (–4.1, 8.2)	0.51
Haplo­type 9 (GTGT)	72	0	79.5 (77.8, 81.1)
	73	1	78.3 (76.6, 80.0)	–1.2 (–2.7, 2.9)	0.34	–0.66 (–2.9, 1.6)	0.57
	43	2	78.1 (76.0, 80.4)	–1.3 (–1.5, 4.0)	0.37	–0.25 (–2.9, 2.4)	0.85
^***a***^Model 1: Arsenic metabolite (%iAs or %DMA or %MMA)=α+β×*N6AMT1* haplo­type+γ×U-As (ln transformed). Model2: Arsenic metabolite=α+β×*N6AMT1* haplo­type+γ×U-As (ln)+λ×*AS3MT* haplo­type2. ^***b***^Number of copies of each haplo­type associated with lower %MMA are denoted first. ^***c***^Mean values and 95% CIs are adjusted values based on model1.							

There were no first-degree relatives but two pairs of second-degree relatives in our study population. In a sensitivity analysis we randomly excluded one in each pair of second-degree relatives and estimated associations between arsenic metabolites and *N6AMT1* SNPs and haplotypes, but results were similar to those reported (data not shown).

Mean %MMA estimated for women with two copies of *N6AMT1* haplotype 1 and two copies of *AS3MT* haplotype 2 was 6.2 (95% CI: 4.6, 7.8), compared with 10.4 (95% CI: 8.7, 12.0) for women with no copies of either haplotype (β = 4.2; 95% CI: 1.9, 6.5 for no copies vs. two copies of each haplotype) (Table 5). This estimated joint association is generally consistent with additive effects of each haplotype, i.e., β = 2.1; 95% CI: 0.75, 3.5 for no copies versus two copies of *N6AMT1* haplotype 1 (Table 4), and β = 3.3; 95% CI: 1.8, 4.7 for no copies versus two copies of *AS3MT* haplotype 2 [see Supplemental Material, Table S3 (http://dx.doi.org/10.1289/ehp.1206003)]. In general, mean %MMA increased as the number of copies of *AS3MT* haplotype 2 copies increased and as copies of *N6AMT1* haplotype 1 decreased, and vice versa, although %MMA was highest among 8 women with either one or two copies of *N6AMT1* haplotype 1 and no copies of *AS3MT* haplotype 2 (11.1; 95% CI: 9.0, 13.2). This pattern of associations was only seen for %MMA.

**Table 5:** Multivariable regression analyses^*a*^ of haplotype × haplotype interaction between *AS3MT* (haplotype 2) and *N6AMT1* (haplotype 1) and fractions of arsenic metabolites.

Metabolite	*AS3MT* haplo­type2+*N6AMT1* haplo­type1^*b*^	*n*	Mean (95%CI)^*c*^	β (95%CI)	*p*-Value
iAs
	2 copies AS3MT+2 copies N6AMT1	15	12.1 (9.1, 15.1)		
	2 copies AS3MT+1 copy N6AMT1	44	11.7 (9.9, 13.5)	–0.42 (–3.9, 3.1)	0.82
	2 copies AS3MT+0 copies N6AMT1	31	10.6 (8.5, 12.7)	–1.5 (–5.1, 2.2)	0.43
	1 copy AS3MT+2 copies N6AMT1	12	13.7 (10.4, 17.1)	1.6 (–2.9, 6.1)	0.48
	1 copy AS3MT+1 copy N6AMT1	30	13.7 (11.6, 15.9)	1.6 (–2.1, 5.3)	0.38
	1 copy AS3MT+0 copies N6AMT1	33	13.2 (11.1, 15.2)	1.0 (–2.6, 4.7)	0.57
	0 copies AS3MT+1 and 2 copies N6AMT1	8	15.6 (11.5, 19.8)	3.5 (–1.6, 8.7)	0.18
	0 copies AS3MT+0 copies N6AMT1	13	15.1 (11.9, 18.4)	3.0 (–1.4, 7.4)	0.18
MMA
	2 copies AS3MT+2 copies N6AMT1	15	6.2 (4.6, 7.8)		
	2 copies AS3MT+1 copy N6AMT1	44	7.2 (6.3, 8.1)	1.0 (–0.75, 2.8)	0.27
	2 copies AS3MT+0 copies N6AMT1	31	8.2 (7.1, 9.3)	2.0 (0.17, 3.9)	0.038
	1 copy AS3MT+2 copies N6AMT1	12	8.0 (6.2, 9.7)	1.8 (–0.55, 4.1)	0.13
	1 copy AS3MT+1 copy N6AMT1	30	9.5 (8.4, 10.6)	3.3 (1.3, 5.1)	0.001
	1 copy AS3MT+0 copies N6AMT1	33	9.7 (8.7, 10.8)	3.5 (1.7, 5.5)	0.00029
	0 copies AS3MT+1 and 2 copies N6AMT1	8	11.1 (9.0, 13.2)	4.9 (2.0, 7.3)	0.00032
	0 copies AS3MT+0 copies N6AMT1	13	10.4 (8.7, 12.0)	4.2 (1.9, 6.5)	0.00041
DMA
	2 copies AS3MT+2 copies N6AMT1	15	81.8 (78.3, 85.3)		
	2 copies AS3MT+1 copy N6AMT1	44	81.1 (79.1, 83.2)	–0.65 (–4.7, 3.4)	0.75
	2 copies AS3MT+0 copies N6AMT1	31	81.1 (78.7, 83.6)	–0.67 (–4.9, 3.6)	0.76
	1 copy AS3MT+2 copies N6AMT1	12	78.3 (74.4, 82.2)	–3.5 (–8.7, 1.8)	0.20
	1 copy AS3MT+1 copy N6AMT1	30	76.8 (74.3, 79.2)	–4.9 (–9.2, –0.57)	0.027
	1 copy AS3MT+0 copies N6AMT1	33	77.1 (74.8, 79.5)	–4.7 (–8.9, –0.47)	0.030
	0 copies AS3MT+1 and 2 copies N6AMT1	8	73.3 (68.5, 78.1)	–8.3 (–14, –2.3)	0.007
	0 copies AS3MT+0 copies N6AMT1	13	74.6 (70.8, 78.3)	–7.2 (–12, –2.1)	0.006
^***a***^Model: Arsenic metabolite (%iAs or %DMA or %MMA)=α+β×*AS3MT* haplo­type/*N6AMT1* haplo­type+γ×U-As (ln transformed). ^***b***^*AS3MT+N6AMT1* haplo­types associated with lower %MMA are denoted first. ^***c***^Mean values and 95% CIs are adjusted values based on the presented statistical model.					

*N6AMT1 expression analyses*. *N6AMT1* expression in whole blood (ILMN_2315569 median = 121 fluorescence units, range 96–160; and ILMN_1754988 median = 112 fluorescence units, range 94–131) was above the overall median level of expression for all transcripts on the array (109.3 fluorescence units, with median expression levels for individual transcripts ranging from 82.9 to 22,445). There were no statistically significant correlations between *N6AMT1* expression and total arsenic concentrations or %MMA (data not shown); the strongest correlation found was between U-As and ILMN_1754988 (*r*_S_ = –0.17, *p* = 0.18). In general, ILMN_2315569 expression was lowest in women with genotypes and haplotypes associated with the lowest %MMA (used as reference group in Table 6), although in all but two cases (rs2205449 and haplotype 1) heterozygous carriers had the highest expression (i.e., there was not a monotonic association according to numbers of alleles). For rs2705671 and *N6AMT1* haplotype 9, there were statistically significant differences among all three genotypes or haplotypes as a group (*p*-values 0.047 and 0.029, respectively), but post hoc analyses showed that the difference for rs2705671 was significant only between TT and GT (*p* = 0.040, compared with *p* = 0.87 for TT and GG); and for haplotype 9 between no and one copies (*p* = 0.027 compared with *p* = 0.94 for 0 and two copies). An allele–dose effect of the expression of *N6AMT1* was observed for some SNPs/ haplotypes (Table 6).

**Table 6:** *N6AMT1* gene expression expressed as relative fluorescence units, stratified by *N6AMT1* SNPs and haplotypes

*N6AMT1* SNP or haplo­type^*a*^	Genotype or no. of copies	*n*	ILMN_2315569	ILMN_1754988
			Mean (95%CI)	Mean (95%CI)
rs1997605	AA	24	117.2 (111.3, 123.0)	111.0 (108.0, 114.0)
	AG	24	126.2 (119.8, 133.9)	114.7 (111.9, 117.8)
	GG	15	122.9 (112.6, 133.2)	115.2 (111.3, 119.1)
		*p* = 0.12^*b*^	*p* = 0.10^*b*^
rs2205449	AA	11	114.7 (107.3, 122.1)	113.9 (108.7,119.1)
	TA	24	120.3 (114.4, 126.3)	112.3 (109.9, 114.7)
	TT	28	126.3 (119.5, 134.2)	114.2 (111.1, 117.5)
		*p* = 0.09^*b*^	*p* = 0.60^*b*^
rs2705671	TT	28	116.8 (111.3, 120.7)	111.9 (108.9, 114.9)
	GT	24	127.3 (120.8, 135.1)	114.6 (111.7, 117.8)
	GG	11	119.6 (107.7, 131.6)	115.7 (111.8, 119.6)
		*p* = 0.047^*b*^	*p* = 0.22^*b*^
rs1048546	GG	21	115.6 (109.3, 121.8)	111.0 (107.5, 114.6)
	GT	28	126.1 (120.3, 132.9)	114.3 (111.8, 117.0)
	TT	14	122.7 (111.6, 133.8)	115.5 (111.3, 119.6)
		*p* = 0.073^*b*^	*p* = 0.15^*b*^
Haplo­type1	2 copies	11	114.7 (107.3, 122.1)	113.9 (108.7, 119.1)
	1 copy	24	120.3 (114.4, 126.3)	112.3 (109.9, 114.7)
	0 copies	28	126.3 (119.5, 134.2)	114.2 (111.2, 117.5)
		*p* = 0.09^*b*^	*p* = 0.60^*b*^
Haplo­type 3	1 and 2 copies	21	125.7 (117.6, 133.7)	111.8 (108.2, 115.4)
	0 copies	42	120.5 (115.6, 125.4)	114.3 (112.2, 116.4)
		*p* = 0.24^*b*^	*p* = 0.20^*b*^
Haplo­type 9	0 copies	27	117.2 (111.9, 121.5)	111.7 (108.8, 114.7)
	1 copy	26	128.1 (121.9, 135.5)	114.1 (111.3, 117.2)
	2 copies	10	119.1 (105.7, 132.4)	116.1 (118.9, 120.4)
		*p* = 0.029^*b*^	*p* = 0.20^*b*^
^***a***^The geno­type/haplo­type associated with lower %MMA is denoted first and used as reference group. rs16983411 was excluded from the analysis because there were so few carriers with variant geno­types (*n*=3). Due to low frequency of carriers of two copies of haplotype3, carriers of one and two copies were merged. ^***b***^*p*-Values from ANOVA.				

## Discussion

In our study population of Andean women, variation in the relative amount of MMA in urine was associated with genetic variation in *N6AMT1* in an allele-dose dependent manner, a finding that supports the hypothesis that *N6AMT1* is involved in human arsenic metabolism. Associations between %MMA and *N6AMT1* variants persisted when adjusted for a common *AS3MT* haplotype that was also associated with %MMA. When homozygous carriers of variants of each gene were compared, associations between *N6AMT1* variants and %MMA were not as strong as associations with the *AS3MT* haplotype. When estimated according to combined copy numbers of *N6AMT1* and *AS3MT* haplotypes associated with low %MMA, associations were consistent with an additive effect of variants in the two genes, such that women with two copies of the two haplotypes had the lowest mean %MMA (6.2; 95% CI: 4.6, 7.8), in contrast with a mean of 10.4 (95% CI: 8.7, 12.0) (β = 4.2; 95% CI: 1.9, 6.5) among women with no copies of either haplotype. Although there were few individuals in some of the combined haplotype groups, the differences observed could be sufficient to increase the risk of arsenic-related disease, as higher %MMA in urine [mostly MMA(V)] is related to increasing risk of several adverse health effects (Chung et al. 2002; IARC 2004; Leonardi et al. 2012; Mazumder et al. 2005; Meliker et al. 2007; Navas-Acien et al. 2005; Vahter 2002). All *N6AMT1* SNPs except one (rs16983411) were common in the study population (minor allele frequencies > 40%) and in HapMap reference populations (Thorisson et al. 2005), and apart from rs2205449, more than half of the participants were carriers of alleles that were associated with lower %MMA. For *AS3MT*, we previously showed a strong overrepresentation of the haplotype associated with low MMA and high DMA (i.e., an efficient and less toxic metabolism) in this Andean population, compared with all other studied populations worldwide (Schlawicke Engström et al. 2007).

The gene *N6AMT1* was recently identified in humans (Ratel et al. 2006) and is located on 21q21.3 [NCBI HomoloGene database (http://www.ncbi.nlm.nih.gov/homologene)]. It is conserved in chimpanzee, dog, cow, mouse, rat, chicken, zebrafish, fruit fly, mosquito, *Caenorhabditis elegans*, *Saccharomyces pombe*, *Arabidopsis thaliana*, and rice (NCBI HomoloGene database). The mouse homolog of N6AMT1 has been shown to methylate glutamine in the translation termination factor eRF1 and to be crucial for embryological development (Liu et al. 2009). N6AMT1 has about 25% amino acid sequence similarity with AS3MT in the *S*-adenosylmethionine binding domain class I [Ajees et al. 2012; see also NCBI Conserved Domains database (http://www.ncbi.nlm.nih.gov/cdd)], a structural fold shared by most methyltransferases (Schubert et al. 2003). However, the structural similarity between N6AMT1 and AS3MT does not include the three cysteine residues (C72, C174, and C224) that bind inorganic AsIII and MMAIII and are necessary for the arsenic methylation steps performed by AS3MT (Marapakala et al. 2012). Thus, other amino acid residues are probably important for the arsenic methylating capacity of N6AMT1. In accordance with the previous experimental studies on *N6AMT1* and arsenic methylation (Ren et al. 2011), our results suggest that %MMA may be influenced by *N6AMT1*. Although the associations with %iAs or %DMA were not statistically significant, the consistent opposite direction of the associations with iAs and MMA, which is in contrast to the findings for *AS3MT* (Engström et al. 2011), suggests different effects of *N6AMT1* on the two methylation steps. Findings for *AS3MT*, on the other hand, suggest that it may affect both steps to about the same extent (Engström et al. 2011).

A limited range of expression of *N6AMT1* in blood may be one of the reasons why we did not find any association between *N6AMT1* expression in whole blood and total arsenic in urine; alternatively, the expression of *N6AMT1* may not be induced by arsenic. Genotypes associated with low %MMA were associated with the lowest *N6AMT1* expression, although in most cases expression did not change monotonically according to allele or haplotype copy numbers. The direction in expression is similar to observations for *AS3MT*: For carriers of the haplotype associated with more proficient arsenic metabolism and less MMA in urine, reduced *AS3MT* expression was found in blood (Engström et al. 2011). Still, the relation between genotype–gene expression and metabolite pattern should be further explored, ideally in tissues where *N6AMT1* is highly expressed, such as the adrenal and parathyroid glands and the kidneys (http://www.proteinatlas.org) (Ren et al. 2011; Uhlen et al. 2005).

Apart from *AS3MT* and *N6AMT1*, we have previously reported evidence that genetic variation in DNA methyltransferases *DNMT1* and *DNMT3B* influences arsenic metabolism efficiency, and similar to *N6AMT1*, associations with these methyltransferases were weaker than associations with *AS3MT* variants (Engström et al. 2011; Gardner et al. 2012). These findings suggest that *AS3MT* is the major arsenic methyltransferase but that other, probably less specific methyltransferases, can also methylate arsenic. In addition, although part of the differences in arsenic metabolism efficiency is genetically determined, the phenotype appears to be polygenic.

## Conclusions

Polymorphisms in *N6AMT1* significantly predicted the %MMA in urine in a population of women from the Argentinean Andes, suggesting additional pathways and methyltransferases involved in the metabolism of arsenic. This emphasizes the need to consider *N6AMT1* in future studies of populations exposed to arsenic.

## Supplemental Material

(561 KB) PDF
