# Supplementary material for: N-6-Adenine-Specific DNA Methyltransferase 1 (N6AMT1) Polymorphisms and Arsenic Methylation in Andean Women
Source: Environ Health Perspect. 2013 May 10;121(7):797–803. doi: 10.1289/ehp.1206003 (PMC3702000; doi:10.1289/ehp.1206003)
Supplement: (561 KB) PDF [file ehp.1206003.s001.pdf]

Supplemental Material

N-6-Adenine-Specific DNA Methyltransferase 1 (*N6AMT1*) Polymorphisms and Arsenic Methylation in Andean Women

Florencia Harari, Karin Engström, Gabriela Concha, Graciela Colque, Marie Vahter, Karin Broberg

Table of contents:

Supplemental Material Table S1. Characteristics of study participants from San Antonio de los Cobres and surrounding villages.

Supplemental Material Table S2. Comparison between study participants recruited 2008 and 2011.

Supplemental Material Table S3. Multivariable regression analyses for arsenic metabolites and *AS3MT* haplotype.

Supplemental Material Figure S1. Linkage disequilibrium-values (R^2) presented as percentages for *N6AMT1* SNPs. Details in Results under "*General characteristics*".

Supplemental Material Figure S2. Error bar plots. MMA fractions in urine (study population, N=188) stratified for *N6AMT1* genotypes of the following SNPs: A) rs1997605, B) rs2205449, C) rs2705671, and D) rs1048546. Error bars represent 95% confidence intervals. Numeric data are provided in Table 3.

Supplemental Material Table S1. Characteristics of study participants from San Antonio de los Cobres and surrounding villages.

	San Antonio de los Cobres (N=154)		Other villages (N=34)		
Characteristics	Mean	P 5th-95th	Mean	P 5th-95th	p-value ANOVA
Age	37	18-64	37	21-63	0.95
Years of Residence	26	5.0-52	25	0.0-53	0.69
BMI	26	19-35	25	18-39	0.22
U-As (µg/L)	277	116-543	59	18-117	<0.001
%iAs	13	4.7-23	13	4.9-25	0.59
%DMA	79	66-89	79	65-90	0.80
%MMA	8,8	4.3-15	7,9	3.3-18	0.15

Supplemental Material Table S2. Comparison between study participants recruited 2008 and 2011.

	San Antonio de los Cobres					Surrounding villages				
	2008 (N=137)		2011 (N=17)			2008 (N=17)		2011 (N=17)		
Characteristics	Mean	P 5th-95th	Mean	P 5th-95th	p-value ANOVA	Mean	P 5th-95th	Mean	P 5th-95th	p-value ANOVA
Age	38	18-64	31	15-50	0.04	35	20-76	40	22-63	0.36
Years of residence	27	5.0-58	22	4.0-50	0.14	21	0.0-53	30	0.5-56	0.12
BMI	26	19-35	26	20-34	0.88	23	17-39	26	19-39	0.06
U-As (µg/L)	284	123-543	215	20-604	0.07	56	14-117	63	22-147	0.55
%iAs	13	4.9-23	10	2.4-23	0.09	15	4.9-29	11	3.7-23	0.11
%DMA	78	66-88	81	64-93	0.14	77	64-90	81	65-92	0.10
%MMA	8.8	4.3-15	8.7	3.7-15	0.89	8.0	3.1-18	7.7	3.3-19	0.81

Supplemental Material Table S3. Multivariable regression analyses[a] for arsenic metabolites and *AS3MT* haplotype.

Metabolite	Statistical Model	*AS3MT* Haplotype[b]	Mean (95% CI)[c]	β (95% CI)	p
iAs	Model 1	2 copies	11.4 (10.2, 12.6)		
		1 copy	13.5 (12.1, 14.8)	2.1 (0.27, 3.9)	0.025
		0 copies	15.3 (12.8, 17.8)	3.4 (1.1, 6.7)	0.0066
	Model 2	2 copies	11.4 (10.2, 12.6)		
		1 copy	13.5 (12.1, 14.8)	2.1 (0.27, 3.9)	0.025
		0 copies	15.3 (12.8, 17.8)	3.9 (1.1, 6.7)	0.0068
	Model 3	2 copies	11.4 (10.1, 12.6)		
		1 copy	13.5 (12.2, 14.8)	2.2 (0.33, 4.0)	0.021
		0 copies	15.3 (12.9, 18.0)	4.1 (1.3, 7.0)	0.0047
	Model 4	2 copies	11.4 (10.1, 12.6)		
		1 copy	13.5 (12.2, 14.8)	2.2 (0.35, 4.0)	0.020
		0 copies	15.3 (12.9, 18.0)	4.1 (1.3, 7.0)	0.0043
	Model 5	2 copies	11.4 (10.1, 12.6)		
		1 copy	13.5 (12.2, 14.9)	2.2 (0.33, 4.0)	0.021
		0 copies	15.3 (12.9, 18.1)	4.2 (1.3, 7.1)	0.0051
	Model 6	2 copies	11.4 (10.1, 12.6)		
		1 copy	13.5 (12.1, 14.9)	2.1 (0.31, 4.0)	0.022
		0 copies	15.3 (12.9, 17.9)	4.0 (1.2, 6.9)	0.0054
	Model 7	2 copies	11.4 (10.1, 12.5)		
		1 copy	13.5 (12.2, 14.9)	2.2 (0.41, 4.1)	0.017
		0 copies	15.3 (13.0, 18.1)	4.2 (1.4, 7.1)	0.0040
	Model 8	2 copies	11.4 (10.1, 12.6)		
		1 copy	13.5 (12.2, 14.8)	2.3 (0.35, 4.0)	0.020
		0 copies	15.3 (12.9, 18.0)	4.1 (1.3, 7.0)	0.004
MMA	Model 1	2 copies	7.4 (6.7, 8.0)		
		1 copy	9.4 (8.7, 10.1)	2.0 (1.0, 2.9)	0.000062
		0 copies	10.7 (9.3, 12.0)	3.3 (1.8, 4.7)	0.000020
	Model 2	2 copies	7.4 (6.8, 8.1)		
		1 copy	9.4 (8.6, 10.0)	1.9 (0.97, 2.9)	0.000095
		0 copies	10.7 (9.3, 11.9)	3.2 (1.7, 4.6)	0.000033
	Model 3	2 copies	7.4 (6.9, 8.1)		
		1 copy	9.4 (8.6, 10.0)	1.8 (0.88, 2.8)	0.00017
		0 copies	10.7 (9.0, 11.7)	2.8 (1.4, 4.3)	0.00016
	Model 4	2 copies	7.4 (6.9, 8.1)		
		1 copy	9.4 (8.6, 10.0)	1.8 (0.89, 2.8)	0.00016
		0 copies	10.7 (9.1, 11.7)	2.9 (1.4, 4.4)	0.00012
	Model 5	2 copies	7.4 (6.9, 8.1)		
		1 copy	9.4 (8.6, 10.0)	1.8 (0.85, 2.7)	0.00025
		0 copies	10.7 (9.1, 11.8)	2.9 (1.4, 4.4)	0.00016
	Model 6	2 copies	7.4 (6.8, 8.1)		
		1 copy	9.4 (8.6, 10.0)	1.9 (0.92, 2.8)	0.00015
		0 copies	10.7 (9.2, 11.8)	3.1 (1.6, 4.5)	0.000061
	Model 7	2 copies	7.4 (6.9, 8.2)		
		1 copy	9.4 (8.6, 10.0)	1.7 (0.80, 2.7)	0.00033
		0 copies	10.7 (9.0, 11.6)	2.8 (1.3, 4.2)	0.00029
	Model 8	2 copies	7.4 (6.9, 8.1)		
		1 copy	9.4 (8.6, 10.0)	1.8 (0.89, 2.8)	0.00016
		0 copies	10.7 (9.1, 11.7)	2.9 (1.4, 4.4)	0.00012

Metabolite	Statistical Model	*AS3MT* Haplotype[b]	Mean (95% CI)[c]	β (95% CI)	p
DMA	Model 1	2 copies	81.2 (79.8, 82.7)		
		1 copy	77.2 (75.6, 78.7)	-4.1 (-6.2, -2.0)	0.00017
		0 copies	74.1 (71.2, 77.0)	-7.2 (-10, -3.9)	0.000023
	Model 2	2 copies	81.2 (79.8, 82.6)		
		1 copy	77.2 (75.6, 78.7)	-4.0 (-6.1, -1.9)	0.00022
		0 copies	74.1 (71.2, 77.1)	-7.1 (-10, -3.8)	0.000032
	Model 3	2 copies	81.2 (79.8, 82.6)		
		1 copy	77.2 (75.6, 78.7)	-4.0 (-6.1, -1.9)	0.00026
		0 copies	74.1 (71.2, 77.2)	-7.0 (-10, -3.7)	0.000050
	Model 4	2 copies	81.2 (79.8, 82.6)		
		1 copy	77.2 (75.6, 78.7)	-4.0 (-6.1, -1.9)	0.00024
		0 copies	74.1 (71.2, 77.1)	-7.1 (-10, -3.7)	0.000041
	Model 5	2 copies	81.2 (79.8, 82.6)		
		1 copy	77.2 (75.6, 78.8)	-4.0 (-6.2, -1.8)	0.00032
		0 copies	74.0 (71.0, 77.2)	-7.1 (-10, -3.7)	0.000056
	Model 6	2 copies	81.2 (79.8, 82.7)		
		1 copy	77.2 (75.6, 78.8)	-4.1 (-6.2, -1.9)	0.00026
		0 copies	74.1 (71.1, 77.1)	-7.1 (-10, -3.8)	0.000036
	Model 7	2 copies	81.2 (79.7, 82.6)		
		1 copy	77.1 (75.6, 78.8)	-4.0 (-6.1, -1.9)	0.000029
		0 copies	74.1 (71.2, 77.2)	-7.0 (-10, -3.6)	0.000058
	Model 8	2 copies	81.2 (79.8, 82.6)		
		1 copy	77.2 (75.6, 78.7)	-4.0 (-6.1, -1.9)	0.00024
		0 copies	74.1 (71.2, 77.1)	-7.0 (-10, -3.7)	0.000041

[a] Multivariable regression Model 1: Arsenic (As) metabolite (%iAs or %DMA or %MMA) = α+ β**AS3MT* haplotype

Model 2: As metabolite = α+ β**AS3MT* haplotype + γ*U-As (ln transformed).

Model 3: As metabolite = α+ β**AS3MT* haplotype + γ*U-As (ln) + λ**N6AMT1* (rs1997605).

Model 4: As metabolite = α+ β**AS3MT* haplotype + γ*U-As (ln) + λ**N6AMT1* (rs2205449).

Model 5: As metabolite = α+ β**AS3MT* haplotype + γ*U-As (ln) + λ**N6AMT1* (rs2705671).

Model 6: As metabolite = α+ β**AS3MT* haplotype + γ*U-As (ln) + λ**N6AMT1* (rs16983411).

Model 7: As metabolite = α+ β**AS3MT* haplotype + γ*U-As (ln) + λ**N6AMT1* (rs1048546).

Model 8: As metabolite = α+ β**AS3MT* haplotype + γ*U-As (ln) + λ**N6AMT1* Haplotype 1.

[b] The haplotype associated with lower %MMA is denoted first and used as reference group.

[c] Mean values and 95% confidence intervals are adjusted values based on the presented statistical model.

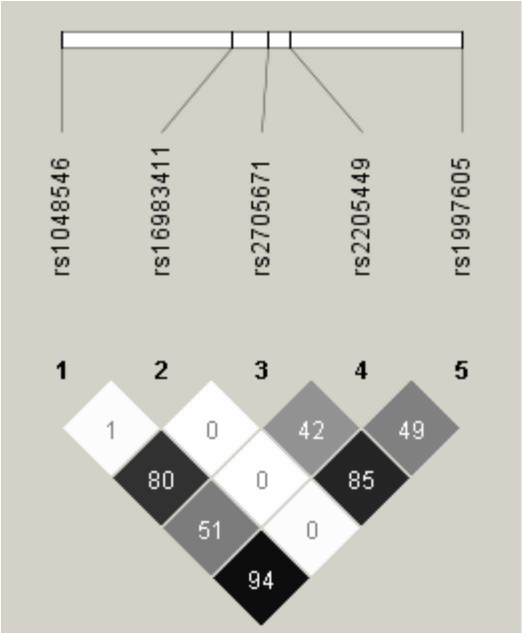

Supplemental Material Figure S1. Linkage disequilibrium-values (R^2) presented as percentages for *N6AMT1* SNPs. Details in Results under "*General characteristics"*.

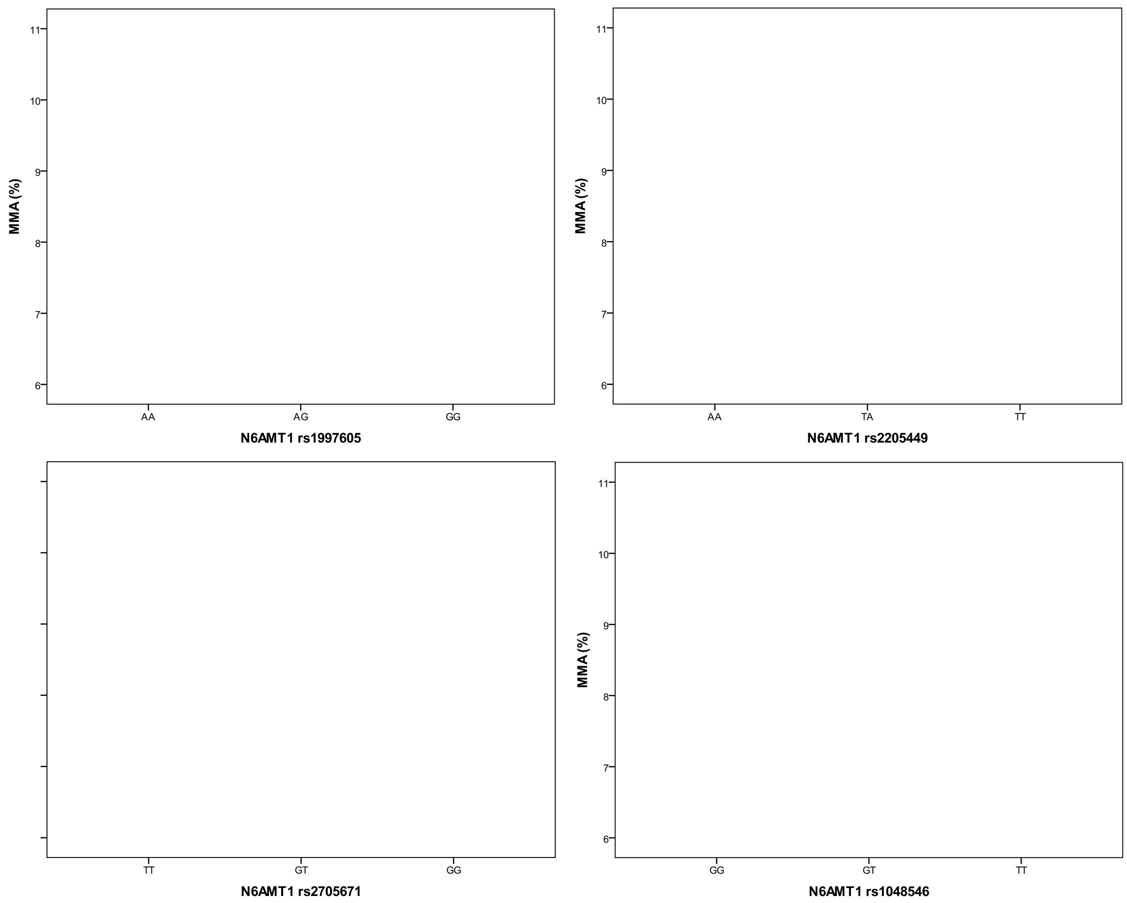

Supplemental Material Figure S2. Error bar plots. MMA fractions in urine (study population, N=188) stratified for *N6AMT1* genotypes of the following SNPs: A) rs1997605, B) rs2205449, C) rs2705671, and D) rs1048546. Error bars represent 95% confidence intervals. Numeric data are provided in Table 3.
